# Factors affecting the performance of community health workers in India: a multi-stakeholder perspective

**DOI:** 10.3402/gha.v7.25352

**Published:** 2014-10-13

**Authors:** Reetu Sharma, Premila Webster, Sanghita Bhattacharyya

**Affiliations:** 1 Indian Institute of Public Health Gandhinagar (IIPHG), Public Health Foundation of India (PHFI), Gujarat, India; 2 Nuffield Department of Population Health, The University of Oxford, Oxford, UK; 3 Public Health Foundation of India, New Delhi, India

**Keywords:** community health workers, ASHA, link workers, child health, NRHM

## Abstract

**Background:**

Community health workers (CHWs) form a vital link between the community and the health department in several countries. In India, since 2005 this role is largely being played by Accredited Social Health Activists (ASHAs), who are village-level female workers. Though ASHAs primarily work for the health department, in a model being tested in Rajasthan they support two government departments. Focusing on the ASHA in this new role as a link worker between two departments, this paper examines factors associated with her work performance from a multi-stakeholder perspective.

**Design:**

The study was done in 16 villages from two administrative blocks of Udaipur district in Rajasthan. The findings are based on 63 in-depth interviews with ASHAs, their co-workers and representatives from the two departments. The interviews were conducted using interview guides. An inductive approach with open coding was used for manual data analysis.

**Results:**

This study shows that an ASHA's motivation and performance are affected by a variety of factors that emerge from the complex context in which she works. These include various personal (e.g. education), professional (e.g. training, job security), and organisational (e.g. infrastructure) factors along with others that emerge from external work environment. The participants suggested various ways to address these challenges.

**Conclusion:**

In order to improve the performance of ASHAs, apart from taking corrective actions at the professional and organisational front on a priority basis, it is equally essential to promote cordial work relationships amongst ASHAs and other community-level workers from the two departments. This will also have a positive impact on community health.

Community health workers (CHW) link communities and health systems in many countries, such as Uganda, Ethiopia, Zambia, Bangladesh, Brazil, to empower communities to demand health services and improve outreach and coverage of health services (1).

India launched its CHW programme in 1977 and then again, in a new form, in 2005 (2). The recent programme was launched as a part of a larger health sector reforms initiative, known as the National Rural Health Mission (NRHM), an umbrella programme of the health department of Government of India (GoI). The cornerstone of NRHM is recruiting one female volunteer as an Accredited Social Health Activist (ASHA) to serve as a health educator and promoter, helping the community access health services. More than 0.8 million ASHAs have already been recruited and placed across India (3). Two other groups of female community-level workers who contribute to child health in India are the Auxiliary Nurse Midwife (ANM), who is with the health department, and the Anganwadi worker (AWW), who is with the department of women and child development (DWCD).

The ASHA is a female village resident, preferably a daughter in-law, with a minimum of 8 years of formal school education. She is expected to serve a population of 700 in tribal areas and 1,000 in rural villages. She is selected by the community-level governance body (Panchayat), its leader (Sarpanch), other villagers, and officials from health and aligned departments at a formal village-level meeting (Gram Sabha) (4). The ASHA undergoes a training of 23 days, conducted under the guidance of the health department (5). The ASHAs are supervised at the primary health centre (PHC) level and receive performance-based monetary incentives from the health department on a monthly basis (4). Her key roles and responsibilities include

1. identifying and registering new pregnancies, births, deaths;
2. mobilising, counselling, and supporting the community to demand and seek entitled health services;
3. identifying, managing, or referring diseased cases;
4. supporting health service delivery through home visits, first-aid, immunisation sessions, and camps; and
5. maintaining data and participating in community-level health planning (4).

Almost all the functions of ASHAs are supportive in nature to ANMs, who are stationed at sub-centres that cover populations of 3,000–5,000 in tribal and rural areas. They are employees of the health department and are paid a monthly salary (6). Each ANM is supported by three to five ASHAs.

This ASHA–ANM joint working model functions in all the states of India. In Rajasthan, ASHAs are also expected to support AWWs who implement the DWCD's Integrated Child Development Scheme (ICDS), a national-level child health, education and nutrition programme. The AWW serves a village population of 700–1,000 through a village-level institution, the Anganwadi centre (AWC) as an ICDS worker and is paid a fixed monthly honorarium by DWCD.

The Rajasthan model differs from other states because prior to the launch of NRHM in 2005, the ICDS in Rajasthan planned a post of a support staff for AWWs. But after NRHM was launched, ICDS merged its support staff position with NRHM's ASHA to avoid duplication. This led to the sharing of ASHAs between the departments of health and DWCD from 2005 onwards. Both departments are expected to participate in the selection, training, monitoring, supervision, and providing incentives for work to ASHAs (7, 8). Thus, while ASHAs are village health volunteers who receive performance-based incentives, the ANMs and AWWs are workers of their respective departments that pay them fixed remuneration on monthly basis.

The multiplicity of community-level workers, their interlinked roles and responsibilities, and the unique arrangement of ASHAs to support both (DWCD and health) departments in Rajasthan inspired the doctoral level study conducted between 2009 and 2014 to understand the coordination between the ASHAs and their co-workers (ANMs and AWWs). This paper is limited to using a multi-stakeholder's perspective to explain the factors that affect ASHA's work performance and strategies that can improve their performance.

## Methods

The doctoral level study used mixed methods while the findings presented in this paper are based on in-depth interviews (IDIs) with ASHAs, their co-workers and health system representatives (DWCD and health department officials).

### Study area

The study was done in 16 villages of two administrative blocks of Udaipur district in Rajasthan. The reason for first selecting Rajasthan and then Udaipur from Rajasthan was that both are classified as high priority zones under NRHM, due to their high infant mortality rate, that is, 63 (Rajasthan) and 62 (Udaipur) against 57 as national rate (9, 10). Moreover, Udaipur has a high tribal population proportion, that is, 46% compared to 12% in the entire state (11). The two administrative blocks selected from Udaipur district are rural. One block has a significant (>80%) tribal population, whereas the other mainly has a rural population (>80%). Thus, the eight villages selected from the tribal block are referred to as ‘tribal’ and the eight villages selected from the non-tribal but rural block are referred to as ‘non-tribal’ in this paper. The selection of 16 villages from the two blocks was based on multi-stage purposive sampling.

From two selected blocks, eight PHCs (four per block) were selected based on their geographical representation of the block and proportion of rural population covered. A total of 16 sub-centres from eight PHCs (two per PHC) were then selected based on their population coverage; availability of ANM; and ANM's years of work experience. Finally, 16 villages from 16 sub-centres (one per sub-centre) were selected based on their population coverage; the availability of AWC, AWW, ASHA; and years of work experience of AWW and ASHA. Census 2001 of GoI and block level data from the two departments was used in this selection.

### Participants

The study participants were ASHAs, ASHA's co-workers, and health system representatives (DWCD and health department officials from PHC/sector, block, and district levels). The insights from ASHAs’ co-workers and health system representatives were important as they observe, supervise, and provide inputs to ASHAs on a daily basis.

The study included one ASHA, AWW, and ANM from each selected village. This is because, as per government norms, a tribal and rural village of 700–1,000 population should have one ANM, one ASHA, and one AWW. The selection of 16 villages, as explained above, ensured that each village had an average specified population size and one ANM, ASHA, and AWW with at least 3 years of experience of working together. A break-up of the study participants is provided in Table 1.

**Table 1:** Stakeholders who participated in the study

Stakeholders		Number of participants
Community workers	ASHA	15
	AWW	16
	ANM	15
Health system	Department of Health	11
representatives	Department of Women and Child Development	6

### Study instrument

The IDIs were based on interview guides. The questions focussed mainly on three broad themes: the experience of joint working between ASHA and co-workers; factors that affect this joint working; and suggestions to improve this joint working. In case the respondent cited the ASHA's poor work performance or support as a reason for poor coordination between the three groups of workers, the study instrument provided scope to probe the reasons for the lack of support and suggestions to improve her performance. The findings presented in this paper are limited to ASHAs’ work performance.

### Data collection and management

All interviews were conducted with informed verbal consent of the study participants. Each interview took an average of 45 min. The majority of interviews were audio-recorded and field notes were used where permission for audio-recording was not granted. All IDIs in audio and notes format were translated from Hindi to English before the final analysis.

### Data analysis

An inductive approach with open coding was used to analyse the data manually, using Microsoft Excel. To avoid any coding bias, each interview was coded independently first. The codes that emerged from all IDIs were compared and codes of a similar nature were grouped and re-grouped into appropriate categories. For example, codes such as education, personal health, family background, and domicile were grouped under ‘personal factors’ category (Table 2).

**Table 2:** Key factors from a multi-stakeholder perspective that effect the ASHA's performance

Keys categories	Self (ASHA), *N*=15	Co-workers (ANM, AWW), *N*=31	Health system representatives (WCD and health department), *N*=17
Personal	Family background	Education Health Family background Domicile	Family background Domicile
Professional	Placement Job security Incentives Relationship with Co-workers	Recruitment Placement Training, knowledge and skills Job accountability Attendance monitoring Job security Incentives Relationship with co-workers	Recruitment Placement Training, knowledge and skills Job accountability Attendance monitoring Job security Incentives Relationship with co-workers
Organisation	Supervision Infrastructure	Supervision Infrastructure	Supervision Infrastructure
External work environment	People and place	People and place	–

### Ethical consideration

The Public Health Foundation of India Institutional Ethics Committee (PHFI, IEC) approved the research. Informed verbal rather than written consent was taken from the study participants because reluctance to sign paper formats was observed amongst participants. Any compulsion for written consent would have biased the data and affected its quality. The majority of interviews were conducted at the residence of the participants to ensure their comfort and confidentiality. The time spent in rapport building with participants prior to the interviews and informing them about steps that will be taken to protect their identity and confidentiality ensured their comfort in sharing information during interviews. The names of selected PHCs, sub-centres, and villages are not mentioned in this paper to ensure this confidentiality. The individual participants were coded and their responses were grouped as responses by ASHAs, co-workers, and health system representatives to ensure confidentiality.

## Results

Table 2 summarises factors that affect ASHA's work performance. They have been classified under four broad categories: personal, professional, organisational, and external work environment.

### Personal

While few ASHAs cited any personal reasons impacting their support to co-workers, about one-third of the co-workers and health system representatives identified factors on the ASHA's personal front that adversely affected such support.

#### Education

Both having more than the required education level as well as less than the minimum level of education needed for this position affect an ASHA's performance. Those with more than the required education were perceived to be less interested in field-based work that fetched limited financial incentives. Those with less than the required education were seen as having limited capacity and knowledge to support their co-workers.

The ASHA is educated up to 12th standard. She is disinterested in the ASHA role and has applied for the ANM's post. This is the main reason for her not working. … Co-worker, Tribal village

My ASHA is less educated than required which is why she is unable to fill basic registers and prepare reports … Co-worker, Non-tribal village

#### Health

With the majority of ASHAs belonging to the reproductive age group (25–40 years), absenteeism from work due to frequent pregnancies and other maternal health issues is a common factor affecting their performance. The absence of any government policy on making interim arrangements during an ASHA's maternity leave affects the health service delivery of the area.

Many ASHAs do not work during pregnancy but this ASHA has left everything. This is creating problem for me … No substitute is provided by government in such cases … Co-worker, Non-tribal village

#### Family background

The structure, caste, norms, and status of ASHA's family were said to affect her work performance. As females, the ASHAs are expected to obey their in-laws and spouse and give priority to domestic over professional work. If her spouse and in-laws belonged to an economically and politically strong background, they were reluctant to let her do field-based work that fetched her less social status and financial incentives compared to her co-workers. Discrimination of ASHAs belonging to lower castes by the co-workers affected her motivation and performance.

Her husband works with Panchayat. He says that she (ASHA) doesn't need this type of job. He says that she can earn Rs 500 just like that. She is afraid of him hence doesn't work … Co-worker, Non-tribal village.

I do not feel like working because I also have to do the household work. ASHA work is exhausting; I am unable to take care of my children … ASHA, Tribal village

#### Domicile

Both being resident and not being the resident of the village they serve were said to affect ASHAs’ performance. Non-resident co-workers felt that resident ASHAs disobeyed them as they felt more powerful and fearless due to support from local political leaders and villagers. On the other hand, many non-resident ASHAs were said to lack interest in their work, often remained absent and lacked rapport with the villagers.

This ASHA is not from our village nor stays here. Hence she does not take interest in working for this village. … Co-worker, Non-tribal village

### Professional

All groups of study participants named one or more of the following professional factors as adversely affecting ASHAs’ motivation and performance.

#### Recruitment

The non-consultative and in-haste selection of ASHAs by Panchayat leaders was held as being responsible for the appointment of politically connected, economically well-off, and sometimes non-resident ASHAs, who were usually relatives, friends or supporters of Panchayat members. Protected by political leaders, they were seen to be less in need of a job and hence more disrespectful to their co-workers, less fearful of retrenchment or punitive action. They were, therefore, often poor performers.

ASHAs who truly need the job perform whereas those that come through politicians don't. No one can compel her to work and if compelled, she creates political pressure. … Health system representative

#### Placement

The ‘sandwiched’ placement of ASHAs between DWCD and the health department in Rajasthan was said to have led to duplication of her work, reporting and supervision, causing wastage of her time and effort, confusion, conflicts and lack of control by either department. Participants from both departments complained of ASHAs’ greater support to the other department and for ignoring their department.

The ASHA is directed to only complete the health department's work by ANMs. The health department people do not allow her to work for AWWs though she should support both departments. … AWW, Non-tribal village

#### Training, knowledge, and skills

The ASHAs’ performance was said to be adversely impacted by their limited orientation on their own and their co-workers’ role; poor training on counselling and health promotion skills; and an over-emphasis on their ‘incentive-based’ rather than ‘activist’ role. The strategy of training ASHAs after they are selected for the post rather than mandating a basic training course as a pre-requisite for applying for the post was said to limit their motivation to learn. All these issues, it was suggested, led to unaccountability, incompetency and, hence, the poor performance of ASHAs.

ASHAs have not been trained the way they should be … They get limited training on community mobilisation, child immunisation and others due to which they have limited knowledge and skills … We are training ASHAs after recruitment rather than asking them to successfully complete a course to apply for this post. The assured job in hand decreases her motivation to learn new things in training. … Health system representative

#### Job accountability

Participants said that expecting ‘support’ from ASHAs in the absence of job clarity or clear job descriptions from the government and lack of clarity amongst ASHAs and co-workers makes it difficult for co-workers to demand ‘support’ or challenge ‘no support’ from ASHAs. This limited role clarity amongst all and absence of role accountability in ASHAs led to her greater non-compliance and poor performance.

There are no clear orders that instruct ASHA to help us in this or that. It just depends on her wish. If she wants she can. If she refuses, the work is left on us. We are also unclear on where to ask for her support and where not. … Co-worker, Non-tribal village

#### Attendance monitoring

Due to the poor monitoring of ASHAs’ attendance the majority of them did not sign their daily attendance at the AWC and did not inform AWWs of their field visits. The approval of forged attendance records of ASHAs by Panchayat leaders, it was pointed out, further encouraged non-performance and non-compliance.

ASHAs don't come to the AWC for days and then the AWW complain to us. This creates conflict between the two workers after which ASHAs stop supporting AWWs … The Panchayat and ANMs also sign false attendance records of ASHAs. No one really care. … Health system representative

#### Incentives

The ASHA incentives from both departments were said to be inadequate, irregular, delayed and paid separately rather than, as ASHAs would prefer, a consolidated amount. Lack of clarity on ‘who will pay’ and ‘how much for what job’ along with no incentives for some tasks (such as patient referrals, data management) affect her motivation and performance. However, few co-workers and health system representatives complained about ASHAs’ participation only in incentivised tasks over others. The DWCDs’ fixed monthly honorarium model of cash payment, it was alleged, made ASHAs consider it their right without performing any duties.

The payment from both departments is very less so we feel de-motivated … The health department do not pay on time and this money is sometimes paid in instalments. … ASHA, Non-tribal village

#### Job security

The absence of a clear career path for ASHAs post-NRHM, lack of clarity on any reward or promotion as a result of their good performance, and not having an ‘employee status’ with any department unlike their co-workers have led to job insecurity and lack of motivation to work amongst ASHAs.

The career path and promotions for AWWs and ANMs within their own departments are certain but not for ASHAs hence they are de-motivated … Health system representative

We don't know what is going to happen in future. We can be removed tomorrow. We are not permanent. We have been allocated work only till 2012. This de-motivates us to work. … ASHA, Tribal village

#### Relationship with co-workers

ASHAs cited instances of their co-workers’ abusive, inconsiderate, unfriendly, oppressive, and biased behaviour (on the basis of class and caste) towards them. They also gave examples of poor work ethics in co-workers such as corruption, greed, cheating, and bribery. This lowered ASHAs’ respect for, and hence support to, co-workers. ASHAs alleged that ANMs were forging financial bills, bribed eligible couples for sterilisation, sought money for free government services from pregnant women and forced ASHAs to share their financial incentives with them. They alleged that AWWs were also forging financial bills and often shifted their own work to ASHAs, giving excuses such as their old age, poor health, limited education, training, and skills.

The AWWs neither behave properly with us nor help us … They fight and scold us over work. … Why should we assist them then? … ASHA, Tribal village

The ANMs ask for their share from our monthly incentives … They also take bribe from pregnant women for delivery … Now we have stopped helping ANMs in many things. … ASHA, Non-tribal village

### Organisational

The majority of ASHAs but only a few co-workers and health system representatives believe that poor supervision and infrastructural support affect their work performance.

#### Supervision

According to study participants, multiple supervisors at the PHC level and ASHAs having to attend monthly meetings and submit reports to both departments cause duplication of work, waste of time and efforts and confusion. The ASHA's morale and performance is also affected by uncoordinated (at times contradictory) instructions by multiple supervisors, lack of joint meetings with both supervisors and the absence of a grievance redressal mechanism.

There is no proper coordination between the supervisors and their instructions to the ASHAs and this results in leaving the task undone and de-motivates ASHAs to work. … Co-worker, Tribal village

#### Infrastructure

The ASHA's performance and motivation is affected by the absence or irregular refurbishment of various job aids, such as medical kits and data entry registers, poor service delivery at the village level due to the lack of staff at health facilities and shortage of vaccines on village immunisation days. The staff shortage and vaccine issues result in low uptake of health services by community members that ASHAs had motivated.

There are no daily diaries for ASHAs … The registers are limited and not renewed regularly … Children are sent back without vaccination due to lack of vaccines on immunisation day … People motivated by ASHAs to seek services from health centres are not attended to properly … Many such things de-motivate ASHAs. … Health system representatives

### External work environment

Participants from all categories discussed the discouraging challenges ASHAs face while working in difficult field situations such as distant, hilly, scattered, inaccessible and insecure geographical locations; combined with illiteracy, myths and misconceptions, religious and cultural beliefs, and fear and distrust of community people on ASHAs and co-workers.

The area is hilly, scattered and dangerous for a woman to work alone. Hence she avoids making home visits … People don't trust ASHA but consult me for everything … ASHAs are also unable to motivate cases for family planning due to illiteracy amongst people in this area … ASHAs refuse to assist ANMs in far off areas. … Co-worker, Tribal village

### Suggestions by participants

In relation to personal factors, participants suggested that the government should set a clear maternity leave policy for all groups of CHWs and a policy on maternity cover for ASHAs. To address other personal factors, suggestions for careful ASHA selection included strict adherence to the educational qualifications; employment contract with a condition to serve for some minimum period; excluding women from influential families; and selection of all groups of community-level workers from the same Panchayat or geographical zone.

“The government must make some interim arrangements for field support for us while the ASHA is on maternity leave” … They must avoid selection of highly educated ASHAs … They should avoid selecting ASHAs who are family members of Panchayat members. Rather those that are poor or widow must be given a chance … They must only appoint the three workers that reside in the same Panchayat or nearby locations. … Co-workers, Tribal and non-tribal village

To address professional issues, participants suggested that the ASHA selection should be either done by one or both departments jointly with the Panchayat, but not by the Panchayat alone. While some participants felt that an ASHA should only be placed with one department, there were several who believed that the ASHAs can perform well even when she is placed with two departments if there is good coordination between the two departments.

Suggestions on issues of training, knowledge, and skills included short-term (5–6 months) pre-recruitment rather than post-recruitment training of ASHAs; improvement in training content; and emphasis on role clarity, counselling skills, data management, and sharing attitude amongst community workers. Participants suggested that both co-workers should jointly certify ASHA's attendance and make surprise field visits while both supervisors should jointly review ASHA's attendance in monthly meetings.

Suggested measures to improve the incentive system in cash and in kind were increased, performance-based, timely and jointly paid incentives; clear career progression path; and awards for good work. To improve inter-worker relationships, supervisor's role in addressing grievances of ASHAs and co-workers and inculcating values of mutual respect and team work amongst all were suggested.

Both departments and the Panchayat together should select ASHA … ASHA should be either put in one department or align all her functions between both departments … The supervisors must make all workers understand that they should work as a team and respect each other to be able to work together. … Co-workers, Tribal and non-tribal villages

Work must be clearly divided between each of us so that we consider our responsibility towards the work. Also, ownership towards the work increases thinking that the particular work is mine … Our payment should be increased from both departments as we work for both. Instead of paying separately it is better if we get paid jointly by them … ASHAs, Tribal and non-tribal villages

ASHAs should be asked to clear a five to six months course prior to applying for the post. … Health system representatives

On organisational issues like supervision, joint supervisory monthly meetings for ASHAs’ work planning, attendance and work monitoring along with measures to improve overall inter-departmental coordination were suggested. On infrastructural issues, timely replenishment of registers, records, medical kits and vaccine supply were suggested to streamline ASHAs’ work. There were no suggestions for improving the physical infrastructure of AWCs and health centres.

Both the department supervisors should take ASHA's joint meetings so that there is no disparity on ASHA related issues. … Health system representative

We are only able to give them (patients) limited medicines. If PHC provides us all the medicines in the medical kit on timely basis, we will be able to serve people better. … ASHA, Non-tribal village

For improving the external work environment, regular joint field visits by ASHA and co-workers to counsel villagers were suggested to improve people's knowledge and rapport with these community workers. Apart from highlighting the need to ensure the security of ASHAs when they travel to distant locations, they suggested additional incentives for ASHAs working in tough terrains.

If we (ASHA, co-workers) counsel village women together then the women will understand the importance of child immunisation and will get their children for immunisation. … ASHA, Non-tribal village

## Discussion

The study showed that the ASHAs’ performance is affected by a variety of factors that emerge from the complex context in which they work. Of the four categories, the challenges on the professional front were most commonly recognised by all the groups of participants. However, the participants’ responses were based on their closeness to the context. For example, challenges related to field work were identified by more ASHAs than the health system representatives, who only review and supervise it as an outsider.

Apart from commonly known personal factors, this study revealed other issues that affect ASHA's performance, such as over-qualification in education; domicile status; and health challenges due to the reproductive age of ASHAs. Of all the personal factors, the link between the socio-economic and political background of the ASHA's family and her performance was cited by many co-workers and health system representatives.

While professional factors (selection, role clarity, training, skills, supervision, incentives, and job security) were highlighted by a number of earlier studies (1, 12–16), this study reveals other factors that can affect a CHW's (ASHAs in this case) job motivation and performance: recruitment based on favouritism rather than competency; lack of clear affiliation of ASHAs to any department/authority; absence of mechanisms to ensure job accountability; and non-cordial, relationship amongst ASHAs and co-workers.

In addition to the organisational factors like supervision and infrastructure that have been identified in past studies (17), this study reveals that geographical and community-level challenges (classified as external work environment here) may also affect motivation and work performance of female community-level workers.

While this study was conducted in the unique context of Rajasthan, many of its findings were similar to those conducted elsewhere in India (18, 19). Similarly a systematic review of CHW programmes from 35 countries of Africa, Asia, Europe, and the United States also showed similar factors affecting the work performance of CHWs. These common factors are: CHW's education, distance of residence from work place, socio-cultural status, training, role clarity, job skills, infrastructure and job support, supervision, incentives, clarity on career progression, affiliation to a government department and perceptions and myths within the client community (1).

Based on the analysis of these factors and lessons learnt from this study, the two concerned departments (health and DWCD) should prioritise corrections in the following areas: ASHA selection, role clarity, training, infrastructure, monitoring, supervision, and incentive systems. Inter-departmental and multi-stakeholder consultations with institutions such as ASHA mentoring groups, the National and State Health Systems Resource Centres and inter-departmental steering groups and committees at the national and state level are required. The purpose of such consultations should be to identify, institutionalise and monitor the consultative process of ASHA selection; develop clear and comparative job descriptions of ASHAs and co-workers; do periodic reviews and up-gradation of ASHA training content, methodology and quality; rationalise and have a periodic replenishment of work aids; design ASHA's attendance monitoring mechanism; study the feasibility of joint reporting and supervision of ASHAs by both departments; and examine the option of direct bank transfer of performance-based incentives to ASHAs jointly by both the departments in Rajasthan. Such multi-stakeholder consultations have the potential of assessing the feasibility of various corrective actions in the ASHA programme and facilitating their conversion to policy directives.

## Conclusion

The study showed that ASHAs work in a complex inter-personal, inter-professional, and inter-organisational environment, in addition to a challenging external geographic and ethnographic environment. Apart from prioritising corrective action at the professional and organisational front, the study identifies the need for improving trust, respect, and rapport between all groups of community workers to enable a ‘Community Health Team’ approach that will not only improve ASHA's performance but also have a positive impact on the overall community's health.
